# Whole-Genome Sequences of Multidrug-Resistant *Escherichia coli* Strains Sharing the Same Sequence Type (ST410) and Isolated from Human and Avian Sources in Italy

**DOI:** 10.1128/genomeA.00757-15

**Published:** 2015-07-09

**Authors:** Maria Giufré, Marisa Accogli, Caterina Graziani, Luca Busani, Marina Cerquetti

**Affiliations:** a Department of Infectious, Parasitic and Immune-Mediated Diseases, Istituto Superiore di Sanità, Rome, Italy; b Department of Veterinary Public Health and Food Safety, Istituto Superiore di Sanità, Rome, Italy

## Abstract

Extraintestinal pathogenic *Escherichia coli* (ExPEC) is involved in a wide spectrum of human diseases. Chickens have been suggested as reservoirs for fluoroquinolone (FQ)-resistant ExPEC strains. Here, we report the whole-genome sequences of 4 *E. coli* strains sharing the same sequence type (ST) (ST410) and that were isolated from human and avian sources in Italy.

## GENOME ANNOUNCEMENT

Extraintestinal pathogenic *Escherichia coli* (ExPEC) is involved in a wide spectrum of human diseases, including urinary tract infections (UTIs), septicemia, and neonatal meningitis (1). The worldwide diffusion of resistance to commonly used antimicrobial agents, such as fluoroquinolones (FQ), trimethoprim-sulfamethoxazole, and β-lactams is a serious public health concern (2). Chicken and chicken products have been suggested to be a source for FQ (often multidrug [MDR])-resistant ExPEC strains causing infections in humans as a result of the massive use of these antimicrobial agents in poultry farming practice (3, 4). In our previous studies, we found that a subgroup of *E. coli* strains belonging to phylogenetic group A are potentially exchangeable between poultry and humans and may constitute a potential zoonotic risk (5, 6). In particular, we identified some closely related multilocus sequence type (ST) clones belonging to specific clonal complexes (CC) (CC10 and CC23, both belonging to phylogenetic group A) that were associated with FQ resistance and multidrug resistance and were shared among strains isolated from UTIs, sepsis, and avian species (5). Among these potentially zoonotic clones, ST410 was frequently detected in human extraintestinal infections occurring in Italy (5).

In this study, we determined the whole-genome sequences of 4 *E. coli* strains, all belonging to ST410 (Table 1). Of these, 2 strains were isolated from human extraintestinal infections (1 from UTI and 1 from sepsis), and 2 strains were isolated from avian species (1 from a chicken with colibacillosis and 1 from a healthy chicken) during the period of April to July 2012.

**TABLE 1:** Accession numbers and assembly statistics for whole-genome sequences of 4 *E. coli* strains

Isolate	Source (infection)	BioSample no.	ST^a^	GenBank accession no.	Genome coverage (×)	Genome size (Mb)	No. of contigs	*N*_50_ (bp)	No. of genes
BE127	Human (UTI)	SAMN03466678	410	LBBB00000000	258	4.86	261	42,079	5,045
Pa1s	Human (sepsis)	SAMN03466682	410	LBBD00000000	60	4.94	237	55,342	4,794
avian5	Avian (commensal)	SAMN03466683	410	LBBE00000000	237	5.00	238	40,982	4,844
46zs	Avian (colibacillosis)	SAMN03466680	410	LBBC00000000	379	4.95	346	28,872	4,732

a ST, sequence type.

Genomic DNAs were extracted from an overnight culture using the NucleoSpin DNA extract kit (Macherey-Nagel, Duren, Germany). Whole-genome sequencing was performed using Illumina MiSeq (250-bp paired-end reads) technology (Illumina, San Diego, CA). The genome sequences were assembled *de novo* using Newbler (7). The resulting coverage ranged from 60× to 379×, with an average of 233×. Genome annotation was performed using Glimmer3 (8) and the NCBI Prokaryotic Genome Annotation Pipeline (PGAP) (http://www.ncbi.nlm.nih.gov/genome/annotation_prok/).

The results of genome sequencing are summarized in Table 1. Overall, the 4 *E. coli* genomes consist of chromosomal DNA, with several plasmids, with an average length of 4.94 Mbp and 4,854 putative protein-coding genes.

Further detailed studies of the reported genomes, including comparative analysis with other available *E. coli* genomes from both human and avian sources, will be reported soon. These genomic data will improve our understanding of the possible spread of FQ (MDR)-resistant *E. coli* clones from chickens to humans that might contribute to the burden of ExPEC infections.

### Nucleotide sequence accession numbers. 

These whole-genome shotgun projects have been deposited at DDBJ/EMBL/GenBank under the accession numbers listed in Table 1. The versions described in this paper are the first versions.
